# Debunking Myths and Misinformation in Cervical Cancer: A Narrative Review on Navigating Complex Treatment Choices in Locally Advanced Cases and Exploring Beyond Standard Protocols

**DOI:** 10.3390/diagnostics15091174

**Published:** 2025-05-06

**Authors:** Mustafa Zelal Muallem, Ahmad Sayasneh

**Affiliations:** 1 Department of Gynecology with Center for Oncological Surgery, Charité—Universitätsmedizin Berlin, Corporate Member of Freie Universität Berlin, Humboldt-Universität zu Berlin, and Berlin Institute of Health, Virchow Campus Clinic, Charité Medical University, 13353 Berlin, Germany; 2 Department of Gynecological Oncology, Guy’s and St Thomas’ NHS Foundation Trust, School of Life Course Sciences, Faculty of Life Sciences and Medicine, King’s College London, Westminster Bridge Road, London SE1 7EH, UK; ahmad.sayasneh@gstt.nhs.uk

**Keywords:** locally advanced cervical cancer, multimodal therapy, chemoradiotherapy, radical surgery, personalized medicine

## Abstract

Cervical cancer remains a significant health challenge globally, with locally advanced cervical cancer (LACC) representing a particularly complex subset due to its diverse definitions and varied treatment approaches. The absence of randomized controlled trials comparing the upfront radical surgery followed by concurrent chemoradiotherapy (CCRT) or chemotherapy alone for clearly defined risk factors for LACC hinders the development of standardized treatment protocols, leading to disparities in patient outcomes across different healthcare settings. This paper seeks to underline the necessity of a consensus on the definition of LACC and aims to comprehensively and critically review the existing literature trying to harmonize treatment strategies and improve prognostic outcomes. Our analysis suggests a multimodal approach for treating FIGO IB3, IIA2, and selected IIB cases at facilities capable of delivering highly curative nerve-sparing surgical interventions, with the goal of bridging the gap in current treatment methodologies. Preliminary findings suggest that adopting a standardized definition could facilitate more consistent treatment outcomes and enhance comparative research.

## 1. Introduction

The definition of locally advanced cervical cancer (LACC) significantly influences treatment approaches and outcomes yet lacks consensus. This inconsistency leads to varied management strategies across different healthcare systems globally. Establishing a standardized definition of LACC is essential to enhance patient care and prognosis.

**The first myth: LACC is a well-defined category of cervical cancer lesions.**

The LACC category traditionally includes patients classified within FIGO Stage IIb to IVA. Recent discussions have expanded this definition to encompass patients with FIGO Stage IB3 and IIA2. The National Comprehensive Cancer Network (NCCN) [1] and ESGO (European Society of Gynecological Oncology) [2] guidelines have very similar definitions of LACC, while some guidelines, such as the Brazilian, Japanese, and Korean guidelines, do not define specifically this group of patients [3].

**Figure 1:**
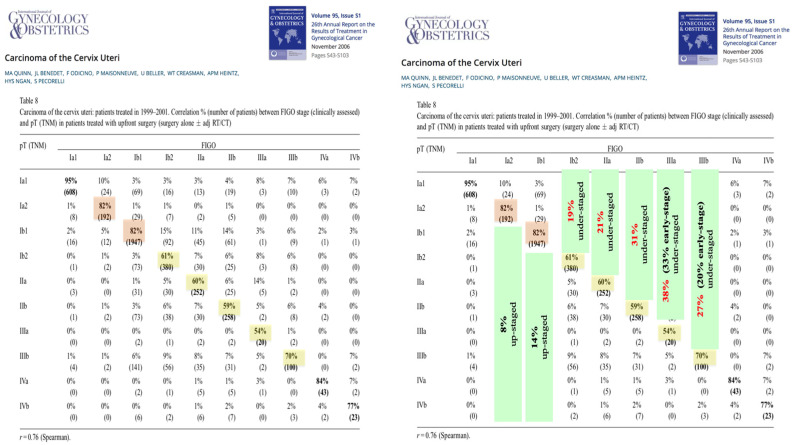
The discrepancy between the clinical staging and the surgical/pathological staging of cervical cancer in the 26th annual report on the outcomes of treatment in gynecological cancer from 2006 [5].

The second issue is that most of the available data on LACC, and consequently in guidelines, relies on the FIGO 2009 classification [4], which is based solely on clinical assessment of the disease. In their 26th annual report on gynecological cancer treatment outcomes from 2006, Quinn et al. detailed the findings from a subset of 6000 out of 15,000 patients. These patients received treatment either through surgery alone or in combination with adjuvant radiotherapy and/or chemotherapy [5]. In Table 8 in their study, they examined the relationship between FIGO stages (which are based on clinical evaluation) and pT stages of the TNM classification (which are based on pathological evaluation). The accuracy of FIGO staging in this large data lay only between 54% and 70% for FIGO (2009) stages IB2 (equivalent to IB3 in FIGO 2018) [6] to IIIB. Nineteen percent of FIGO stage IB2 cases (equivalent to IB3 in the FIGO 2018 classification) are downstaged after surgical therapy. This happens in 21% of stage IIA cases, 31% of stage IIB cases, 38% of stage IIIA cases (33% even to an early stage I–IIA), and 27% of stage IIIB cases (20% to an early stage). This means that about 20–40% of patients who are clinically assessed as having LACC are denied surgical therapy only because of the high bias of clinical classification. On the other hand, only 8–14% of stages IA2 and IB1 cases (equivalent to IB1 + IB2 in FIGO 2018) are upstaged after surgery. Figure 1 shows this discrepancy between the clinical staging and the surgical/pathological staging of cervical cancer in the 26th annual report on the outcomes of treatment in gynecological cancer from 2006.

1. *Fact: LACC is not clearly defined. As is evident from the preceding, clinicians have a tendency to upstage cervical cancer through clinical examination. This can disqualify 20–40% of clinically staged patients from surgery. These findings highlight the need to improve diagnostic tools to accurately identify patients with LACC who would benefit from surgical therapy. At present, surgery followed by clinicopathological evaluation remains the best method for stratifying patients at risk who may require adjuvant therapy.*

**The second myth: Standard therapy for LACC is the definitive chemoradiotherapy, aimed at avoiding multimodal therapeutic approaches.**

The 2021 FIGO cancer report stated that ”Although feasible, surgery as initial treatment in FIGO Stage IB3 and IIA2 is not encouraged for patients with Stage IB3 and IIA2 disease since 80% of them require radio- or radiochemotherapy” [7].

The authors cited only the Landoni study in reference to the 80% requirement for adjuvant therapy [8]. This study, which randomized patients with early-stage (IB or IIA) cervical cancer to surgery with or without postoperative radiotherapy (PORT) or to definitive radiotherapy alone, has a number of flaws and shortcomings, which can be summarized in the following points:

1. The study was a single-center study conducted between September 1986 and December 1991 and was concurrent with his other single-center study [9], which was conducted between April 1987 and December 1993 and compared radical hysterectomy for class II versus class III in the same group of patients (early cervical cancer with stage IB-IIA).
2. The Landoni study was designed for early-stage cervical cancers in FIGO stages IB–IIA, not for LACC; however, its findings are frequently cited in discussions on LACC treatment.
3. The study aimed to compare surgery with or without postoperative radiotherapy (PORT) against definitive radiotherapy alone. It was not designed for subgroup analyses of patients in the surgery arm who had risk factors and consequently received adjuvant radiotherapy, in contrast to the definitive radiotherapy group, in which 40–60% of cases were expected to have no risk factors. The study’s conclusion that the combination of surgery and radiotherapy leads to the worst morbidity, particularly urological complications, is unwarranted. Complication rates for grade 2 and grade 3 were 31% and 33% in the surgery-only subgroup, compared to 29% and 24% in the surgery plus radiotherapy subgroup (*p* = 0.71) for tumors <4 cm and >4 cm, respectively. This suggests that the majority of complications were linked to surgical procedures themselves. The high complication rate following radical hysterectomy in early-stage cervical cancer aligns with the high rate of cut-through tumors in the surgery group (11%; 6% for tumors <4 cm and 22% for tumors >4 cm), raising concerns about the quality of surgery in this study.
4. Five years later, Landoni was the last author of a multicentric Italian study comparing neoadjuvant chemotherapy and radical surgery with definitive radiotherapy in LACC [10]. In this multicenter study, 29% of patients receiving surgery also underwent adjuvant radiotherapy. Notably, the authors observed no significant increase in severe morbidity across the treatment arms, even with the application of all three modalities to a subset of the surgery group. This finding prompts critical questions about the necessity and design of further studies incorporating multiple therapy modalities, especially given previous evidence suggesting an elevated complication rate with such combinations. Moreover, the absence of a marked rise in severe morbidity, despite the inclusion of chemotherapy, warrants deeper investigation. In a comparative context, the Gupta trial—a more recent study—analyzed outcomes of neoadjuvant chemotherapy (NACT) followed by radical surgery versus concomitant radio- and chemotherapy (CCRT) in patients with stage IB2 (FIGO 2009, equivalent to IB3 in FIGO 2018), IIA, or IIB squamous cervical cancer. This study revealed that 43.6% of patients in the surgical arm received adjuvant radiotherapy. Interestingly, the CCRT group experienced a higher complication rate compared to the NACT + surgery group, particularly concerning long-term complications (>24 months). This difference was markedly significant for vaginal/sexual complications, with rates of 19.9% versus 36.9% (*p* < 0.001) for NACT + surgery versus CCRT 90 days post-therapy, and 12% versus 25.6% (*p* < 0.001) for NACT + surgery versus CCRT 24 months post-therapy [11].

2. *Fact: The combination of more than one therapy modality by treating cervical cancer with risk factors may increase morbidity; however, we know from other randomized controlled trials (GOG92 [12] and GOG109 [13]) that it improves survival outcomes, which justifies its application in high-risk patients. The data from the Landoni study could not be used to refute the use of multimodal therapy in patients with LACC or those with early-stage cervical cancer with high risk, as the study was not designed to answer this question. In the Landoni study, the comparison of the group with definitive primary radiotherapy without any risk factors in the 40–60% range to the subgroup of surgery with adjuvant radiotherapy, which has at least one proven high-risk factor, represents a measurement bias.*

The 2021 FIGO cancer report stated that combined treatment modalities unnecessarily overburden the inadequate surgical and radiation facilities in low-resource countries [7]. We believe that, in the era of personalized medicine, it is difficult to accept an argument that prohibits patients with a good performance status in high-resource countries from receiving the most effective multimodal therapy concept. Even when discussing developing countries, where 85% of cervical cancer cases occur [14], the rigorous schedule and the high costs of CCRT must be considered. Studies estimate the costs of CCRT at USD 22,320 (USD 1518 for chemotherapy, USD 10, 407 for whole pelvic radiotherapy, and USD 10,395 for brachytherapy) without adding the costs of travel, lodging, and outpatient medication, which place a disproportionately heavy burden on women of lower socioeconomic status, even in the United States [15]. The comparison between definitive radiotherapy and adjuvant radiotherapy is frequently ignored; the dose of definitive radiotherapy (the total combined dose with external beam radiotherapy (EBRT) and intracavitary radiotherapy (ICRT)) should be in the range of 80 Gy for small-volume cervical tumors or ≥85 Gy for larger-volume cervical tumors, which is twice that of adjuvant radiotherapy (where a dose of 45–50 Gy is standard) [1].

The combination of two modalities must therefore be understood as:

1. A stratification tool for patients with high risk after surgery by clear definition (pathologically) of the risk factors, thereby sparing any further therapy (in 40–60% of cases initially appearing as early-stage cases and 20–40% of those initially suggested to have locally advanced tumors).
2. A useful tool to treat cancers that are likely to be resistant to radiotherapy.
3. Reducing the applied radio- or radiochemotherapy by fifty percent, thereby sparing a significant number of post-radiation complications, particularly long-term complications, and avoiding the high costs of unindicated CCRT.
4. Offering the opportunity to conserve ovarian function.
5. Improving survival results (this point will be discussed in detail later).

**The third myth: CCRT achieves the best outcomes in LACC:**

To the best of our knowledge, there are no randomized controlled trials comparing primary radical hysterectomy followed by adjuvant therapy with primary CCRT. In spite of this, most guidelines and the majority of authors suggest primary CCRT as standard therapy, citing studies not designed to answer this question. The 2021 FIGO cancer report stated, for example, that the prognosis in terms of overall survival, progression-free survival, and local and distant recurrences is more favorable with CCRT than with radical hysterectomy followed by radiotherapy as postoperative adjuvant therapy [7].

The report cited two studies for this statement: the first is the GOG 109 study [13], which compares adjuvant radiotherapy with adjuvant CCRT, and the second is a GOG randomized study comparing concurrent single-agent cisplatin, cisplatin-based combination chemotherapy, or hydroxyurea during pelvic irradiation for locally advanced cervical cancer (no surgery) [16].

The Gupta trial, comparing NACT followed by radical surgery with CCRT in patients with stage IB2 (FIGO 2009, equivalent to IB3 in FIGO 2018), IIA, or IIB after excluding patients with positive lymph nodes from the surgery arm, reported a 5-year DFS in the neoadjuvant chemotherapy plus surgery group of 69.3%, compared with 76.7% in the CCRT group (hazard ratio, 1.38; 95% CI, 1.02 to 1.87; *p* = 0.038), whereas the corresponding 5-year OS rates were similar in both groups (75.4% and 74.7%, hazard ratio, 1.025; 95% CI, 0.752 to 1.398; *p* = 0.87). This study showed increased delayed toxicities 24 months or later after treatment completion in the CCRT group: rectal (3.5% vs. 2.2%, for CCRT vs. NACT + surgery, respectively), bladder (3.5% vs. 1.6%, respectively), and vaginal (25.6% vs. 12%, respectively) [11].

Most randomized controlled trials on CCRT have reported 3-year OS around 65% [17,18], and the same or worse rates have been reported in retrospective trials [19,20,21,22,23,24,25].

Studies with very low rates of locally advanced cervical cancer show better results (3-year OS of 83% when including only bulky FIGO IB tumors [26] and 5-year OS of 74% in the EMBRACE I study, with only 15.2% of cases in stage III) [27]. These results are clearly inferior to the results of the GOG 109 trial, in which patients received adjuvant radiochemotherapy after radical surgery due to positive lymph nodes (FIGO IIIc), parametrial infiltration (FIGO IIB), or cutting through the tumor (5-year overall survival: 80%) [13].

The most recent randomized controlled trials comparing CCRT with CCRT and targeted therapy like PD-1 inhibitors are in line with the abovementioned results when examining the CCRT arm only. This negates the myth that the current CCRT enhances the outcomes reported in the old studies. The CALLA trial reported that 2-year progression-free survival was 62.1% for CCRT only. Serious adverse events occurred in 89 patients (23%) who received CCRT only [28]. The most recent Keynote A-18 trial showed a 2-year progression-free survival of 57.3% with CCRT only. The incidence of grade 3–4 treatment-related adverse events was 60% in the CCRT group [29]. The final analysis of this study, published very recently, reported a 36-month overall survival of 82.6% in the pembrolizumab–chemoradiotherapy group and 74.8% in the placebo–chemoradiotherapy group [30].

In the real world, primary CCRT seems to deliver worse outcomes depending on many factors, especially treatment time. Song et al. reported that the 3-year OS for all patients was 66% (95% confidence interval [CI], 9%) and that for patients with stage I, II, and III was 60%, 71%, and 62%, respectively (*p* = 0.2). The 3-year DFS for all patients was 58% (95% CI, 9%), and for patients with stage I, II, and III disease, it was 57%, 67%, and 43%, respectively (*p* = 0.01) [31].

In a large retrospective study, where the National Cancer Database (NCDB) was queried to identify stage II–IVA cervical cancer patients diagnosed in the United States between 2004 and 2015 who were treated with definitive chemoradiation therapy, 10,172 women were identified. Only 59.4% of them received brachytherapy, and only 2978 (29.3%) completed treatment within 8 weeks (standard of care = SOC). This emphasized that even in a high-income country with very good facilities like the United States, the rigorous regime of CCRT remains a challenge, with only 29.3% of patients completing it [32].

3. *Fact: CCRT still shows only poor outcomes in LACC. The median 3-year OS is only around 65% when taking the randomized controlled trials into account. For LACC, there is an unmet need for seeking new multimodal therapeutic concepts, including targeted therapy and even surgery.*

**The fourth myth: All high-risk factors are an indication for adjuvant chemoradiotherapy.**

Positive lymph nodes (a regional risk factor that is resectable operatively), microscopic involvement of the parametrium (a local risk factor that is resectable operatively), and positive margins (a local risk factor with operative failure) are the high-risk factors that necessitate adjuvant radiochemotherapy, according to the GOG 109 trial [13].

Note that grouping local and regional, surgically resectable, and surgically failed risk factors together is not the best way to figure out how important these risk factors are for prognosis or whether adding adjuvant therapy will improve the prognosis.

To evaluate the effect of adjuvant treatment in patients with high-risk cervical cancer after radical hysterectomy, the Austrian Gynecologic Oncology Group conducted a prospective, randomized, multicenter clinical trial between 1989 and 1995 to treat 76 patients with stage IB–IIB (parametrial involvement) cervical cancer with radical hysterectomy [33].

Patients who showed pelvic lymph node metastases and/or vascular invasion were randomly assigned to receive adjuvant chemotherapy (400 mg/m^2^ carboplatin and 30 mg bleomycin), standardized external pelvic radiation therapy, or no further treatment. After a median follow-up of 4.1 years (range, 2–7) there were no statistically significant differences in disease-free survival among the three treatment arms, and the authors concluded that radical abdominal hysterectomy with systematic pelvic lymphadenectomy appeared to be the most effective treatment for these patients. Even though this study had some weaknesses, such as the small number of patients recruited and the old chemotherapy and radiotherapy regimens, it is still significant because it is the only randomized controlled prospective study comparing adjuvant therapy with no further therapy in such a cohort of patients with high-risk factors.

The 5-year overall survival rate was 86% in the chemotherapy arm, 81% in the no further therapy arm, and 80% in the radiation arm. In addition, at least four retrospective studies on patients with high-risk cervical cancer have concluded that patients without adjuvant treatment did not fare worse than those with adjuvant treatment [34,35,36,37].

The current standard of care for these patients is based on the findings of the GOG 109 trial, which compared concurrent radiochemotherapy with radiotherapy alone, but not with no additional therapy, and concluded that the addition of chemotherapy to radiotherapy significantly improves progression-free and overall survival for high-risk, early-stage patients undergoing radical hysterectomy and pelvic lymphadenectomy for carcinoma of the cervix [14].

In the radiotherapy-only arm and the CCRT arm, the hazard ratios for progression-free survival and overall survival were 2.01 (*p* = 0.003) and 1.96 (*p* = 0.007), respectively. The projected progression-free survival rates at four years were 63% for radiotherapy only and 80% for CCRT. At four years, the projected overall survival rate was 71% for radiotherapy alone and 81% for CCRT. According to the authors, the favorable survival rate observed in patients receiving the third and fourth cycles of chemotherapy after the completion of radiotherapy suggests that the chemotherapy has an effect independent of the radiotherapy and is not merely a radiation sensitizer.

These ideas were supported and emphasized by the findings of a study conducted on a large population cohort using the National Cancer Data Base (NCDB) to evaluate postoperative high-risk patients with cervical cancer for factors associated with a benefit from chemoradiotherapy compared to external beam radiation therapy alone [38].

After analyzing the data from 3053 eligible patients, the authors concluded that the use of adjuvant chemoradiation therapy after hysterectomy improves overall survival in patients with high-risk cervical cancer compared with EBRT alone; however, this benefit appears to be limited to nodal-positive patients. Chemoradiation therapy did not improve overall survival in patients with only positive margins (*p* = 0.73), only parametrial invasion (*p* = 0.95), or any combination of these two factors in the absence of lymph node involvement (*p* = 0.63). These findings may allow us to conclude that the local risk factors (involvement of the parametrium and/or positive resection margins) benefit more from radiotherapy, whereas the regional risk factor (affected lymph nodes) benefits more from chemotherapy.

The superior efficacy of chemotherapy in patients with affected lymph nodes was confirmed in the multicenter phase II JGOG 1067 trial. In this study, patients with cervical cancer and lymph node metastases were treated postoperatively with irinotecan and nedaplatin. Within six weeks of surgery, chemotherapy was administered and repeated every 28 days for up to five cycles. In this study, the five-year OS rate was 86.5% [39].

In comparison with the GOG 109 study, the 5-year overall survival was 80% in the squamous cell cancer group with adjuvant radiochemotherapy and only 69% in the group with radiation only. These results were validated in the phase III randomized trial of adjuvant chemotherapy versus adjuvant radiochemotherapy for postoperative cervical cancer (JGOG 1082) [40].

4. *Fact: The category of cervical cancer with high-risk factors is a heterogenic group. Accumulating evidence shows that adjuvant chemotherapy, not adjuvant chemoradiotherapy, is the standard of care following surgery in cases of node-positive cervical cancer. However, adjuvant chemoradiotherapy may still be indicated in cases of parametrium invasion and/or incomplete tumor resection (cutting through the tumor).*

**The fifth myth: Abandoning radical hysterectomy is the standard of care when intraoperative detection of positive lymph node metastases occurs.**

The 2018 guidelines for the management of patients with cervical cancer, developed by three European societies (the European Society of Gynecological Oncology, the European Society for Radiotherapy and Oncology, and the European Society of Pathology), recommend sentinel lymph node biopsy and frozen section pathological assessment as the first step in the management of all patients scheduled for primary curative surgery. If sentinel lymph node involvement is found intraoperatively, the guidelines recommend abandoning radical procedures and referring the patient for definitive chemoradiation [41].

The ABRAX trial [42], which was an international, multicenter, retrospective cohort study, attempted to answer this question through retrospective analyses of 515 patients with cervical cancer (51 institutions, 19 countries) referred for primary curative surgery between 2005 and 2015 (stage IA–IIB, common tumor types), in whom lymph node involvement was detected intraoperatively. Patients were stratified according to whether the planned uterine surgery was completed (COMPL group, N = 361) or abandoned (ABAND group, N = 154) to compare progression-free survival. The risk of recurrence (hazard ratio [HR] 1.154), pelvic recurrence (HR 0.836), or death (HR 1.064) did not differ significantly between the two groups. Therefore, the authors concluded that if intraoperative lymph node involvement is confirmed, abandoning the uterine radical procedure and referring the patient for definitive chemoradiation should be considered. The only exception was a marginally increased risk of recurrence among patients with stage IIB tumors in the ABAND group (HR = 2.270 (1.055–4.884), *p* = 0∙036). The ABRAX trial did not include post-treatment complications in either arm; therefore, we cannot determine whether both study arms were similar in terms of short- and long-term complication rates.

In our opinion, the correct interpretation of these data is:

1. There is no survival difference between the primary radical surgery followed by adjuvant radiochemotherapy and definitive radiochemotherapy in early-stage cervical cancer. This is consistent with the findings of the Landoni study, which found no distinction between primary radiotherapy and primary surgery for cervical cancer in its early stages [8]. The completion of a radical hysterectomy and lymphadenectomy reduces radiation exposure (by about 50%) without appearing to compromise safety or outcome, it must be stated [43,44].
2. In locally advanced tumors, the hazard ratio was in favor (significant difference) of primary surgery with adjuvant CCRT.

It is crucial to note that the best prospective randomized controlled trial data available for patients with nodal-positive cancer are from GOG 109 (surgery followed by CCRT) and from JGOG 1067 (surgery plus chemotherapy). Therefore, we believe that the recommendation to terminate the surgery if intraoperatively positive lymph nodes are discovered is unwarranted.

A very recent published study reviewed 2963 patients with positive lymph nodes in 2009 FIGO stage I–III cervical cancer from SEER database and found that radical surgery plus postoperative adjuvant radiochemotherapy improved cancer-specific survival and the overall survival in stage I–II cervical cancer and when there were only five or fewer affected lymph nodes. However, no significant difference was observed between the two treatment modalities in patients with stage III and PLNs > 5 subgroups [45]. Therefore, the authors concluded that a comprehensive assessment of lymph node metastases and local tumor spread should guide rationalized treatment options when managing patients with nodal-positive cervical cancer.

5. *Fact: There is no evidence from randomized controlled trials to support abandoning surgically possible radical hysterectomy only because of the detection of affected lymph nodes. The retrospective studies are controversial, but the largest retrospective study using the SEER database showed improved cancer-specific and overall survival after completing surgery.*

Table 1 summarizes the mentioned myths and the corresponding facts and provides a brief explanation to enhance the clarity of our discussed points.

In conclusion, we share the opinion of other authors around the world [46,47,48] that the treatment outcome of locally advanced cervical cancer is still unsatisfactory. A new therapeutic strategy involving multimodal treatment at a facility that can provide highly curative surgical treatment is desired. Surgery in locally advanced cases is feasible and can be performed nerve-sparingly in more than 90% of cases [49] to minimize postoperative complications if the surgical team has sufficient anatomical knowledge and the right technique to deal meticulously with the parametrium, the paracolpium, and the components of inferior hypogastric plexus [50,51,52].

As we conclude our exploration into the multifaceted decisions surrounding the treatment of locally advanced cervical cancer (LACC), it becomes evident that the journey is as complex as it is critical. The variance in treatment modalities, from surgery to adjuvant therapies, underscores a landscape filled with both promise and caution. Our analysis, drawing upon the insights from recent studies including the pivotal Gupta trial, sheds light on the nuanced outcomes associated with combining treatment modalities. The findings not only challenge conventional wisdom but also pave the way for a more individualized approach to care.

The marked difference in complication rates, especially concerning long-term morbidity and specific complications such as vaginal/sexual health, prompts a re-evaluation of how treatments are selected and combined. It questions the rationale behind adding more therapy modalities without a clear benefit in reducing severe morbidity, highlighting the need for a balance between aggressive treatment and quality of life.

As we move forward, it is clear that the path to optimizing treatment for LACC is not through a one-size-fits-all approach but through a tailored strategy that considers the complexity of each patient’s condition and their specific needs. The journey toward improving outcomes in cervical cancer treatment is ongoing, and each study and data point brings us closer to understanding how best to navigate this challenging landscape. In doing so, we honor the individual stories of those battling LACC, aiming for not just survival but a life lived with dignity and quality.

## Figures and Tables

**Table 1:** **Myths and facts about LACC**.

Myths	Facts	Brief Explanation
LACC is a well-defined category of cervical cancer lesions	There is no unified definition; guidelines vary	Clinical staging may lead to upstaging, disqualifying 20–40% from surgery inappropriately
Standard therapy is definitive chemoradiotherapy to avoid multimodal treatment	Multimodal treatment increases morbidity but improves survival	The Landoni study, often cited here, does not directly compare the correct patient subgroups and introduces bias
CCRT achieves the best outcomes in LACC	3-year OS remains at only ~65%; need for improved multimodal approaches	No RCT compares CCRT to surgery followed by adjuvant therapy in high-risk LACC
All high-risk factors warrant adjuvant chemoradiotherapy	High-risk group is heterogeneous; chemotherapy alone may suffice in node-positive cases	Evidence from NCDB and JGOG 1067 suggests selective use of chemoradiotherapy
Abandoning radical hysterectomy after detecting positive nodes is standard	No RCT supports this; retrospective studies suggest completing surgery improves outcomes	SEER-based data show better survival when surgery is completed, despite nodal involvement
